# Medication Safety Risks and Their Management in Finnish Care Units: A Cross-Sectional Survey

**DOI:** 10.1177/11786329261472941

**Published:** 2026-07-23

**Authors:** Anu Saavalainen, Marianne Kuusisto, Carita Linden-Lahti, Emilia Laukkanen, Tero Vahlberg, Anna-Riia Holmström

**Affiliations:** 1 Division of Pharmacology and Pharmacotherapy, Faculty of Pharmacy, 124454 University of Helsinki, Helsinki, Finland; 2 Finnish Centre for Client and Patient Safety, Wellbeing Services County of Ostrobothnia, Vaasa, Finland; 3 HUS Pharmacy, 525094 Helsinki University Hospital, Helsinki, Finland; 4 4345 Savonia University of Applied Science, Kuopio, Finland; 5 Department of Biostatistics, Faculty of Medicine, 60654 University of Turku, Turku, Finland

**Keywords:** medication safety, patient safety, risk management, healthcare quality improvement, healthcare quality

## Abstract

**Introduction:**

Deficiencies in medication safety are a major concern for patient safety. In Finland, health and social care units are mandated to develop unit-based protocols for safe medication management and use (MMU) to address medication safety risks.

**Objectives:**

The aim of this study is to examine how unit-based safe MMU protocols are developed in Finnish health and social care settings and how comprehensive the content of these protocols is.

**Design:**

A cross-sectional, descriptive study.

**Methods:**

An online survey was conducted among Finnish registered nurses and pharmacists working in health and social care settings. Descriptive statistics were used to summarize the data, and statistical tests (t-tests, Mann-Whitney U, Kruskal-Wallis H) were applied to examine group differences. Sum variables were created to assess protocol content comprehensiveness. The study was conducted and reported in accordance with the STROBE guidelines for cross-sectional studies.

**Results:**

The survey included 905 participants (90.2% nurses and 9.8% pharmacists). Most respondents (91.5%) reported having a unit-based MMU protocol. Only 11.6% indicated that the protocol was developed through interprofessional collaboration, which was more common in healthcare than in social care. Overall, respondents considered the protocols comprehensive, with no major differences between work environments. The least detailed areas were medication safety management (mean 3.09), medication reconciliation and patient involvement (mean 3.13), and risk management in the MMU process (mean 3.30).

**Conclusions:**

Unit-based safe MMU protocols are key tools in describing the MMU process and medication safety practices in Finnish health and social care. However, greater emphasis is needed on interprofessional development, particularly in social care settings, with increased involvement of physicians and pharmacists. Preventive, system-based medication safety risk management needs to be strengthened in the protocols and the competencies of healthcare professionals in the present area.

## Introduction

Medication safety-related risks, such as polypharmacy or high-risk situations (e.g., pediatric medication treatment or transitions of care), are central factors affecting global patient safety in health systems.^1-3^ Such risks may contribute to medication errors, including incorrect dosages or routes of administration. Proactive risk management, grounded in the identification of unit-specific risks in health and social care settings, has the potential to prevent many of these errors.^1^ Introducing system defenses within the units’ medication management and use (MMU) process reduces the possibility of error and patient harm.^2,3^

Safe MMU practices, such as identifying organizational high-alert medications, represent an example of a system defense mitigating the risk of errors.^2,4^ It has been shown that implementing safe MMU practices can be assisted with national programs and recommendations.^5^ In Finland, a National Guideline for Safe Medication Management and Use targeted for health and social care units has existed since 2006. The most recently updated version of the guideline was published in February 2021 by the Ministry of Social Affairs and Health.^6^ The guideline recommends that care units (e.g., hospital wards or nursing homes) develop an internal protocol for safe MMU practices. The protocol is mandated to be updated at least annually (please see the Supplemental Material 1 for the recommended contents of the protocol).

The unit-based safe MMU protocol has been mandatory in Finland for healthcare organizations since 2010 and for social care organizations since 2014 (Act on the Supervision of Social Welfare and Health Care 741/2023). The goal of the protocol is to ensure the safety of the MMU process by describing the safety-promoting practices at the respective care unit.^7^ The protocol should include a description of the MMU process from prescribing to treatment monitoring, the responsibilities of different professionals, the identified unit-specific risks (e.g., high-alert medications used at the unit), and strategies for managing the risks (e.g., implemented defenses to prevent errors in the use of high-alert medications). Protocol should be developed in interprofessional collaboration, and it must be primarily approved by the responsible physician of a unit or by another authorized physician. To our knowledge, such a nationwide, statutory mandated protocol aiming to provide systems-based medication safety promotion of care units has yet to exist in other countries.

Although unit-based safe MMU protocols are a statutory tool for ensuring medication safety in health and social care units, the academic research on their content and impact on medication safety is limited. This study aimed to investigate the use of unit-based safe MMU protocols to promote medication safety in the Finnish health and social care service system. The more specific objectives were to investigate how interprofessional the process of developing the protocols is in units and how comprehensive the protocols are in terms of measures to promote medication safety in different work environments. From an international perspective, the study seeks to provide insights on how such an innovation could be scaled and adopted for proactive nationwide promotion of medication safety in other countries.

## Methods

### Study Design

The study was conducted as a cross-sectional online survey in late 2021 and was reported in accordance with the STROBE (Strengthening the Reporting of Observational Studies in Epidemiology) guidelines. The STROBE checklist is provided as a supplementary file (Supplemental material 2).

The target group comprised registered nurses (RN) (incl. public health nurses, midwives, and paramedics) and pharmacists working in Finland’s health or social care sector (e.g., wards, clinics or nursing homes). These professionals were selected as target groups because they are typically involved in the development of unit-based safe MMU protocols.^8^ Both employees and managers from these professional groups were invited to respond, as well as students if a health or social care unit employed them at the time of the study.

**Figure 1.**
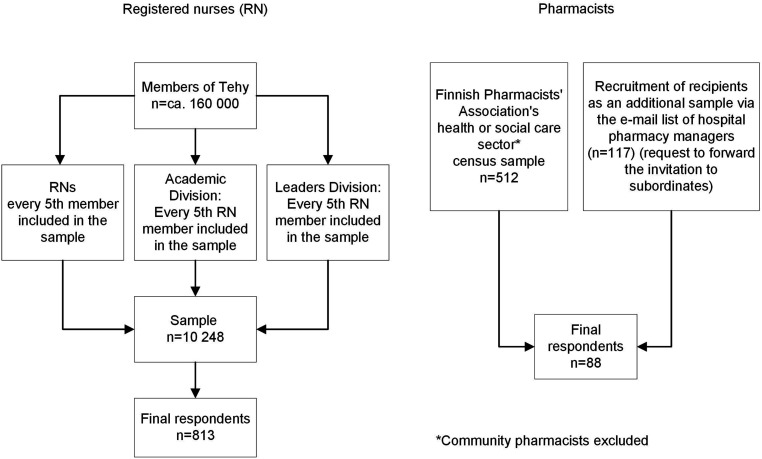
Forming the sample of respondents for the survey

The study sample was formed from the membership registers of the Union of Health and Social Care Professionals in Finland (Tehy) and the Finnish Pharmacists’ Association (SFL) (Figure 1). No formal a priori power analysis was conducted. The sampling strategy was based on maximizing coverage and accessibility of the target populations. For registered nurses, a systematic random sampling approach was used to obtain a large and broadly representative sample, whereas for pharmacy professionals, a census approach was applied due to the smaller and more defined population, supplemented by additional recruitment to improve coverage.

The sample of RNs (n=10 248) from Tehy (ca 160 000 members representing, e.g., RNs, practical nurses, physiotherapists, and early childhood education staff) was formed by randomly selecting every fifth RN member from the register and every fifth RN member of Academic and Leader divisions of Tehy. Retired professionals were excluded from the study. Tehy removed any overlaps between the groups from the sample. In the case of the SFL, a census sample (n=512) was taken of all members who reported working as a pharmacist in health or social care sector (e.g., wards, clinics or nursing homes). The number of respondents (n=34) remained low in the original SFL sample; therefore, additional respondents were recruited by email through managers (n=117) representing Finnish hospital pharmacies. A contact information register maintained independently by hospital pharmacies served as a source of email addresses of the approached managers. Due to the multi-stage sampling process of the pharmacy professionals, the researchers did not have access to the final number of pharmacists invited to participate in the survey. Consequently, the size of the final sample remained unknown to the pharmacy professionals.

### Survey Instrument

An online questionnaire was employed as a survey instrument (Supplemental material 3). The research group developed the questionnaire based on the theoretical foundation of the systems approach to medication safety risk management, the National Guideline for Safe Medication Management and Use recommendations, and the previous research.^3,6,8,9^ The questionnaire included areas identified as currently developing in the field of medication safety, as defined in the most recent version (2021) of the National Guideline for Safe Medication Management and Use.^6^ Conversely, it excluded areas regarded as nationally established components of unit-based safe MMU protocols, based on earlier versions of the guideline (2006 and 2015). These excluded areas encompassed, e.g., descriptions of the operational and personnel structure of unit, as well as internal processes for ensuring staff competence in high-risk procedures (e.g., intravenous administration). In addition, pharmaceutical logistics was excluded from the questionnaire. The sections, themes, and question types of the questionnaire are described in Table 1.

**Table 1.** Structure of the Questionnaire

Section of the questionnaire	Content of the questions (n=25)	Response scale	Respondents
1. Characteristics of respondents	Information related to professional background and working organization	Multiple choice; open text field	All
2. Background information and practices for developing the unit-based safe MMU protocol	Practices for developing the unit-based safe MMU protocol in the unit of the respondent	Multiple choice; open text field	Respondents who participated in developing unit-based safe MMU protocol at their unit
	Professional groups that participated in developing the protocol	Multiple choice	
	Information on how well the staff follows the safe MMU protocol and the instructions included in the protocol	5-stage Likert scale (strongly agree, agree, neither agree nor disagree, disagree, strongly disagree)	
3. Content of the unit-based safe MMU protocol	How are the different practices described in the unit-based safe MMU protocol of unit?	4-stage Likert scale (fully described, partially described, planned to be described, not described, I don’t know)	Respondents who participated in developing unit-based safe MMU protocol at their unit
	The importance of the unit-based safe MMU protocol in developing medication safety at the unit	Open-ended questions	
	Challenges in implementing unit-based safe MMU protocol		
	The support needed by the unit to implement measures that promote medication safety.		
4. Implementation of MMU practices	How the safe MMU practices are implemented in the unit	5-stage Likert scale (excellent, good, fair, poor, very poor)	All
	How does the respondent perceive the level of medication safety culture in their unit	5-stage Likert scale (excellent, good, fair, poor, very poor)	

MMU=Medication management and use.

The questionnaire was piloted to assess the face and content validity in November 2021. A group of healthcare professionals, including RNs (n=6) and pharmacists (n=4), attended the pilot. The pilot group comprised both managers and employees, as well as those who took part in developing the unit-based safe MMU protocol and those who did not but participated in the implementation of the practices described in the protocol at their own unit. Based on the pilot, the questionnaire was subjected to minor technical modifications, such as more specific instructions for some questions and clarifications to the structure of some statements.

The link to the online questionnaire was administered to respondents by email through Tehy, SFL, and hospital pharmacy managers in November-December 2021. The aim and target groups of the survey were explained in the information letter provided to the respondents. The total response time to the survey was two weeks, a reminder was sent one week after the first invitation to participate.

### Data Analysis

The present article reports the results of the questionnaire sections 1-3 (questions 1-19; Supplemental material 3), excluding the open-ended questions (section 3; questions 20-22; Supplemental material 3). IBM SPSS Statistics for Windows (version 28, IBM Corp., Armonk, NY) was employed for statistical analysis. Data was described with n (%), mean (standard deviation), or median (interquartile range) when appropriate. The chi-squared test was used to examine differences in dichotomous variables between groups. The statistical differences were assessed through the t-test for normally distributed variables, the Mann-Whitney-U test, and the Kruskal-Wallis test for non-normally distributed variables. A statistically significant level was set as p<0.05.

The answer options for the 4-stage Likert scale (question 19; Supplemental material 3) were combined into two scales for analysis due to a few extreme responses compared to the data size. The total sum variable was formed from the statements describing the content of the unit-based safe MMU protocol (n=20). In addition to the total sum variables, five subtotal sum variables were theoretically generated. The sum variables were compiled for those respondents who had responded to at least 70% of the statements on that topic. The Cronbach’s alpha coefficient was used to review the internal consistency of sum variables.

## Results

### Participants

A total of 905 professionals responded to the online questionnaire. A small proportion of responses (n=4, <1.0%) were excluded because of deficiencies in the responses (e.g., the respondent had answered only the section on the characteristics of the respondent).

Most respondents (90.2%; n=813/901) were RNs (response rate 7.9%, n=813/10 248) and 9.8% (n=88) pharmacists (total N of population and response rate unknown; Table 2). In the sample of the SFL, the response rate remained low (n=34/512; 6.6%). In the additional sample conducted among hospital pharmacy chiefs and managers (n=117; the final number of recipients to whom the survey was forwarded is unknown), 54 responses were received. The background data of the respondents are described in Table 2.

**Table 2.** Characteristics of the Respondents (n=901)

Characteristic	n	%
**Education**		
Registered nurse	813	90.2
Bachelor of Pharmacy	78	8.7
Master of Pharmacy	10	1.1
Students	0	0
**Time from graduation**		
(Mean 28 years)	​	​
<10 years	49	5.4
10-19 years	64	7.1
20-29 years	359	39.8
30-39 years	394	43.7
≥40 years	29	3.2
N/A	6	0.7
**Position at work**		
Manager	87	9.7
Employee	814	90.3
**Gender**		
Women	855	94.9
Men	34	3.8
Other/choose not to answer	9	1.0
N/A	3	0.3
**Age**		
(Mean 55 years)	​	​
<30 years	11	1.2
30-39 years	35	3.9
40-49 years	96	10.7
50-59 years	513	56.9
≥60 years	243	27.0
N/A	3	0.3
**Working sector**		
Health care	712	79.0
Social care	183	20.3
N/A	6	0.7
**Specific work environment**		
University Hospital	222	24.6
Central hospital^a^	232	25.7
Primary health care ward	126	14.0
Primary health care centre	73	8.1
Social care housing service	133	14.8
Home care	38	4.2
Other^b^	75	8.3
N/A	2	0.2
**Location of the workplace**		
Southern Finland	329	36.5
Western Finland	241	26.7
Northern Finland	136	15.1
Eastern Finland	130	14.4
Middle-Finland	62	6.9
N/A	3	0.5

^a^Includes state hospitals and regional hospitals.

^b^Comprises e.g., emergency care, child welfare, freelance work.

### Awareness and Development of the Unit-Based Safe MMU Protocol

Most of the units of the respondents (91.5%, n=824/901) possessed a unit-based safe MMU protocol. Some respondents were unaware of the existence of such a protocol (5.5%, n=50) or reported missing a protocol from their own unit (3.0%; n=27) (Table 3). Half (48.5%, n=380/784) of the respondents whose unit had a safe MMU protocol and who reported that they were familiar with it had taken part in developing the protocol. Over half (66.8%, n=254/380) of the unit-based safe MMU protocols were updated in 2021.

**Table 3.** Existence of Practices for Developing a MMU Protocol at Units of the Respondents (n=901)

Characteristic	n	%
**Existence of a protocol at the unit of respondents**		
**(n=901)**		
Protocol exists	824	91.5
The protocol does not exist	27	3.0
Does not know	50	5.5
**The respondent’s most recent familiarization with the protocol**	​	​
**(n=824)** ^a^	​	​
Within a year	580	70.4
Over a year ago	134	16.3
Over two years ago	70	8.5
The respondent is not familiar with the protocol	40	4.9
**Own participation in developing the protocol**	​	​
**(n=784)** ^b^	​	​
Has participated	380	48.5
Has not participated	404	51.5
**Time since the latest update of the protocol**	​	​
**(n=380)** ^c^	​	​
<1 year	254	66.8
One year	74	19.5
≥2 years	24	6.3
Does not know	28	7.4

^a^Respondents who have a protocol in their unit.

^b^Respondents whose units have a protocol and who are familiar with the protocol.

^c^Respondents whose units have a protocol, who are familiar with the protocol, and who have participated in developing the protocol.

MMU=Medication management and use.

**Figure 2.**
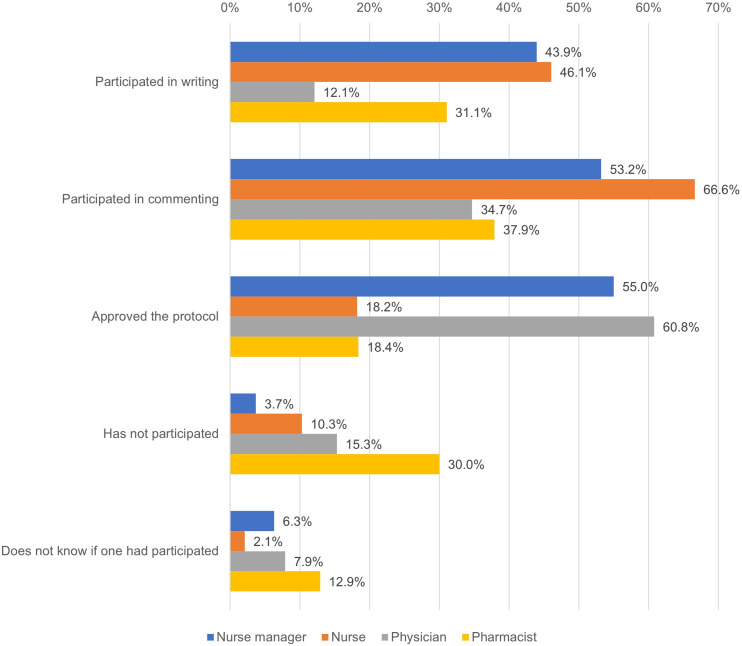
Professionals participating in developing protocols at units of respondents (n=380) who participated in protocol development

Nursing managers and staff were most involved in writing and commenting on the unit-based safe MMU protocol (Figure 2). Pharmacists in writing the protocol were more common than physicians’ involvement. In contrast, the participation of pharmacists and physicians in commenting was almost equal. The pharmacists had most commonly taken part in writing and commenting activities at the university (writing 39.8%, commenting 25.7%) and central hospitals (writing 32.2%, commenting 27.8%), while in other work environments, their participation was lower (e.g., primary healthcare ward: writing 12.7% and commenting 16.0%; social care housing service: writing 3.4% and commenting 16.0%). Approval of the unit-based safe MMU protocol was most often issued by a physician and/or a nursing manager. In answers describing the participation of pharmacists, not participating at all or does not know whether one had participated in developing the protocol was more common than in responses describing the participation of other professionals. However, most responding pharmacists (n= 68/88, 77.3%) had participated in developing the unit-based safe MMU protocol.

The study also assessed how interprofessional the writing, commenting, and approval of the protocol had been in the units of the respondents who participated in developing the unit-based safe MMU protocol (n=380). The process was defined as interprofessional if at least three of the four different professional groups (manager, nursing staff, physician, and pharmacist) had taken part in writing and commenting in the unit of respondents. Only at 11.6% (n=44) of the units, writing a unit-based safe MMU protocol was interprofessional, and 33.9% (n=129) had reached interprofessional participation in commenting on the protocol during its development. Interprofessional participation was more common in health care units than in social care units both for writing (17.6 % vs. 8.3 %, p=0.045) and commenting (48.4% vs. 20.8%, p<0.001) the protocol.

### The Content of the Unit-Based Safe MMU Protocol

For the respondents (n=380) who took part in the development of the unit-based safe MMU protocol, the comprehensiveness of the content of the protocol was assessed using the sum variable formed from the responses of 357 respondents (respondents who had responded to at least 70% of the statements in the respective topic). The sum variable described how well the respondents estimated that the safe MMU practices asked in the survey are described in their unit’s protocol (Table 4; Supplemental material 3, question 19). On average, the respondents estimated that the contents of the protocol are comprehensively described with a mean of 3.33 (scale 1-4, SD 0.56). According to the respondents, the best-described sub-area in the protocols was prescribing, dispensing, and administration practices of medicines (mean 3.50, SD 0.59). Correspondingly, the weakest described sub-areas were the management of medication safety (mean 3.09, SD 0.95), medication reconciliation and patient involvement (mean 3.13, SD 0.81).

**Table 4.** The Comprehensiveness of Content of the Safe MMU Protocols at Units of the Respondents (n=357)^a^

Sum variables and the variables forming the sum variable (areas described in the protocols)	Mean	Standard deviation	Min	Max	Cronbach’s alpha
**Total sum variable**	​	​	​	​	​
Coverage of the content of the unit-based safe MMU protocol in terms of safe MMU practices (n=357)	3.33	0.56	1.00	4.00	0.89
**Subtotal sum variable**	​	​	​	​	​
Competence, training, and orientation in MMU (n=358)	3.36	0.57	1.00	4.00	0.70
• Tasks and responsibilities of MMU					
• Competence required for the implementation of MMU					
• Implementation of MMU orientation					
• Provision of in-house training in MMU					
Risks associated with the MMU process (n=358)	3.30	0.68	1.00	4.00	0.79
• Risks associated with the MMU process of unit					
• Risk management tools					
• High-alert medications in the unit					
• Risk management of high-alert medications					
• Operation in incident situations					
Prescribing, dispensing, and administration practices (n=355)	3.50	0.59	1.00	4.00	0.80
• Prescribing practices					
• Medicines dispensing and compounding or preparation practices					
• Double-checking policies for medicines					
• Medication administration practices					
• Patient identification methods					
• Monitoring the effects of medication treatment					
Medication reconciliation and patient participation (n=356)	3.13	0.81	1.00	4.00	0.79
• Medication reconciliation policies and responsibilities at the patient entry stage					
• Medication reconciliation policies and responsibilities at patient transfer					
• Provision of medication counseling and guidance					
• Supporting patient participation in own medication treatment					
Management of medication safety^b^ (n=356)					
• Monitoring the implementation of measures to promote medication safety at the unit	3.09	0.95	1.00	4.00	NA^b^

^a^The sum variables were compiled from the responses of those respondents who had answered at least 70% of the statements in the respective sub-area (see Supplemental material 3, question 19).

^b^The average of the subtotal sum variable has been calculated based on the responses to only one statement (Monitoring the implementation of the unit’s medication safety; see Supplemental material 3, question 19). In the present case, it is not possible to determine the value of Cronbach’s alpha because it contains only one statement.

MMU=Medication management and use.

The coverage of the unit-based safe MMU protocol was rated most comprehensive in primary care wards and least comprehensive in central hospitals, but the difference was not statistically significant (Supplemental material 4). No statistically significant differences were observed between work environments for subtotal variables (Table 4). Managers (n=64, md=3.60, IQR=0.65) rated the content of the protocol more comprehensive than employees (n=293, md=3.45, IQR=0.77, p=0.048), and nurses (n=289, md=3.50, IQR=0.75) considered their protocol more comprehensive than pharmacists (n=68, md=3.28, IQR=0.62, p=0.005).

**Figure 3.**
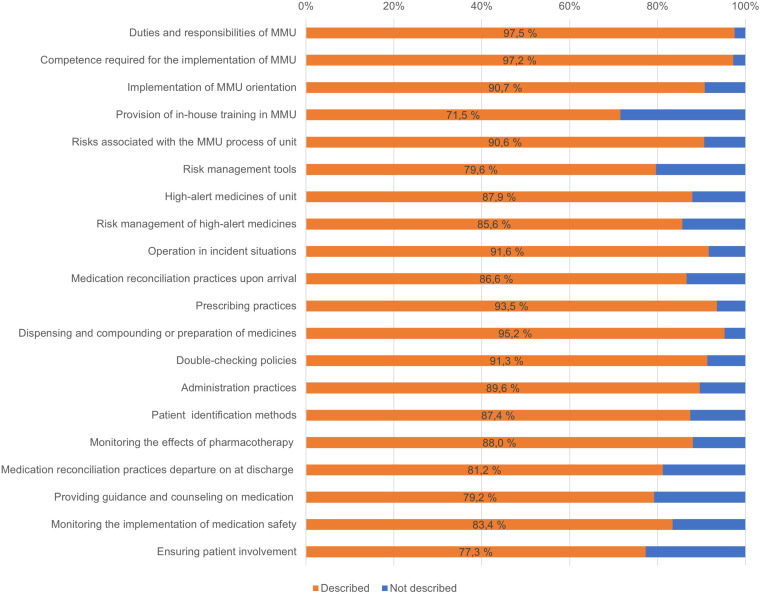
Areas described in protocols of respondents (n=380^a^) who participated in protocol development. ^a^ Not all the respondents answered all statements. MMU=Medication management and use

Means and standard deviations were calculated for individual statements to assess response weights and deviations (Supplemental Material 3, question 19; Supplemental Material 5). According to the individual statements, the area’s most comprehensively described in the unit-based safe MMU protocols were the roles and responsibilities of different professionals in MMU (97.5%) and the competencies needed to implement MMU (97.2%) (Figure 3). The weaker areas described were the organizing of in-house training related to MMU (71.5%) and providing support to patients to participate in their own medication treatment (77.3%).

Respondents involved in developing the unit-based safe MMU protocol were also asked to rate how well they thought the staff was following the unit-based safe MMU protocol and the related guidelines. Most respondents responding to the respective question (93.7%, n=355/379) perceived that the staff adhered well to the unit-based safe MMU protocol. No statistically significant differences were observed between work environments (p=0.631). Managers (n=72, md=5.00, IQR=1) rated the staff better adherent to unit-based safe MMU protocol than employees (n=307, md=4.00, IQR=1, p=0.042), whereas nurses (n=311, md=5.00, IQR=1) rated better adherence than pharmacists (n=68, md=4.00, IQR=0, p=<0.001).

## Discussion

This study indicates that the National Guideline for Safe Medication Management and Use and the development of unit-based protocols have become well known in the Finnish health and social care units; the finding is in accordance with the previous small-scale domestic studies.^9,10^ The present achievement has been preceded by almost 20 years of systematic national initiatives led by the Finnish authorities and their vision of unifying and standardizing safe MMU practices in care units across the country. During this time, the unit-based safe MMU protocol has become a well-known national tool for managing and developing medication safety at local health service system levels. Consequently, the engagement of the national authorities and their understanding of systems-based risk management may represent a key success factor for establishing such a nationwide medication safety initiative. Based on these experiences, the possibility for other countries to adopt a similar protocol should be investigated to facilitate national standardization of medication safety risk management practices.

Even though developing a unit-based safe MMU protocol is legally mandated in Finland, there are still individual units where the protocol has not been developed, or some representatives of key professional groups are unaware of its existence at their units. This finding may reflect that the obligation to develop a unit-based safe MMU protocol may not be known, or if a protocol has been developed, it is not actively used in preventive risk management. More effective utilization of the protocols would require better knowledge and understanding of system-based medication safety risk management for managers and staff. Indeed, previous studies have shown that poor staff education about medication safety and weak safety culture are key factors hindering the effective implementation of medication safety practices.^5,11,12^ Also, the organization’s management may need to have a stronger role in monitoring the existence and quality of protocols to facilitate their effective use.

### The Comprehensiveness of the Protocols

Regardless of the health or social service sector, the respondents perceived the contents of their unit-based safe MMU protocols comprehensively. In practice, however, there may be differences between the description of safety initiatives and their actual level of implementation; this area should be studied more, as well as the quality of the protocol contents. When investigating the individual areas of the unit-based safe MMU protocols, the description of prescribing, dispensing, and administration practices of medicines was reported most comprehensive. On the contrary, the description of practices for managing medication safety, medication reconciliation, patient involvement, and identifying risks related to the MMU process were most limited. This could indicate that describing the MMU process may be easier for the units than describing and implementing the methods used for managing safety risks in the MMU process; the latter requires specialized safety competence and system-oriented thinking. Consequently, these competencies need to be strengthened to standardize the processes in units, which have been found effective in improving medication safety.^13^

Overall, employees and pharmacists were more critical than managers and other professional groups in their assessments of the comprehensiveness of and staff adherence to their units’ protocol. While employees and pharmacists, who often engage most closely in MMU processes and safety management actions, may possess the most realistic view on the status of their units’ protocol, the strong representativeness of these groups needs to be ensured in developing and implementing the protocols. This will also most likely contribute to the creation of a common vision of medication safety risk management and safety culture, as well as support protocol adherence at the unit.^14^

### Interprofessional Process of Developing the Protocols

Interprofessional participation in the development of unit-based safe MMU protocols was found low, although interprofessional teamwork have been shown to promote medication safety.^5,15-18^ Especially the participation of physicians in developing the unit-based safe MMU protocols was limited, which is in accordance with the findings of previous studies.^8,9^ In this study, physicians most often participated in the approval of the protocol, which in practice, can comprise only the technical assignment of the protocol document. However, our results suggest that even this may not be always the case as only 60% reported physicians as professionals approving the protocol at their units. This finding is central from an authoritative perspective, as the National Guideline for Safe Medication Management and Use outlines that the protocol should be primarily approved by the physician responsible for the MMU of the unit or another authorized physician.^6^ Only in units where medication treatment is not daily or demanding (e.g., use of narcotics) can a registered healthcare professional approve the protocol. The factors contributing to the current situation are likely complex but may include physicians prioritizing clinical work over medication safety initiatives, time constraints, professional hierarchies, and adaptation to an organizational culture that does not support preventive risk management.^19^

In the future, it will be necessary to investigate physicians’ knowledge and views regarding unit-based safe MMU protocols in more detail. More importantly, the root causes of their low participation in medication safety activities should be explored, along with means to strengthen their role in effective risk management.

The present study suggests that the interprofessional process of developing the protocols, as well as the role of pharmacists, needs to be increased, especially in primary and social care. The identified more common participation of pharmacists in developing the protocols in university and central hospitals is most likely explained by the fact that most pharmacists in public sector work in these environments, while most primary and social care facilities are currently lacking pharmacy professionals in Finland. The availability of pharmacy expertise in primary and social services should be increased as the pharmacist participation in medication use process has been found to promote medication safety.^11,20-22^ Also, in those units where a pharmacist is available, their expertise may be more likely to be used as more than 75% of the pharmacists who responded to the present survey had participated in developing a unit-based safe MMU protocol. The suggested strategies for increasing the participation of pharmacists in medication safety risk management have been found to comprise interprofessional training, promoting awareness of the role and competencies of different professional groups, as well as support from physicians and the administration of the organization.^23^

### Limitations

The main limitations of the study relate to challenges encountered during the sampling process and the impact of Covid-19 pandemic at the time, both of which likely contributed to the low response rate. A formal a priori power analysis was not conducted, which should be considered a limitation of this study. Additionally, the nationwide shortage of nurses in Finland during the data collection may have further affected participation. Low survey response rates are also a common issue in contemporary research.^24,25^

It was also not possible to determine the sample’s representativeness about the entire population of nurses and pharmacy professionals working in the Finnish public and private health and social care sector, as such data was not available in the scope of this study. Nevertheless, the sample was large enough to enable statistical analysis and describe the current strengths and areas needing development in the use of unit-based safe MMU protocols as a systems-based defense in medication safety in the Finnish health and social care service system.^26^ It is also possible that professionals with a particular interest in protocols and/or medication safety were more likely to respond to the survey. However, this may also represent a strengthening factor to the study, as these professionals have most likely possessed the most comprehensive view of the protocol in their respective unit.

## Conclusions

Unit-based safe MMU protocols are key tools in describing the MMU process and practices in medication safety risk management in the entire Finnish health and social services system. However, the interprofessional development of the protocols should be increased, especially in social care units, and by promoting the involvement of physicians and pharmacists in all units. In the future, staff competencies in preventive, systems-based medication safety risk management may need to be strengthened to establish more comprehensive measures for promoting medication safety. Finally, the possibility for other countries to adopt a similar protocol should be investigated to facilitate national standardization of medication safety risk management practices.

## Supplemental Material

Supplemental Material - Medication Safety Risks and Their Management in Finnish Care Units: A Cross-Sectional Survey Supplemental Material for Medication Safety Risks and Their Management in Finnish Care Units: A Cross-Sectional Survey by Anu Saavalainen, Marianne Kuusisto, Carita Linden-Lahti, Emilia Laukkanen, Tero Vahlberg, Anna-Riia Holmström in Health Services Insights

## Data Availability

The datasets analyzed in this study are available from the corresponding author upon reasonable request.*

## Appendix

### Abbreviations

MMU: Medication management and use
RN: Registered nurse
SFL: Finnish Pharmacists’ Association
Tehy: The Union of Health and Social Care Professionals in Finland
