# Supplementary material for: Medication Safety Risks and Their Management in Finnish Care Units: A Cross-Sectional Survey
Source: Health Serv Insights. 2026 Jul 23;19:11786329261472941. doi: 10.1177/11786329261472941 (PMC13396577; doi:10.1177/11786329261472941)
Supplement: Supplemental Material - Medication Safety Risks and Their Management in Finnish Care Units: A Cross-Sectional Survey [file sj-zip-1-his-10.1177_11786329261472941.zip › Supplementary_material1.docx]

Supplementary material 1. The national recommended content of a unit-based safe medication management and use (MMU) protocol (1)

| 1. Development of the unit-based safe MMU protocol (e.g., includes a short description of the practices of and professionals involved in the development of the protocol at the unit) 2. Implementation of MMU and demandingness level of medication use at the unit    1. Description of the operational and the work unit    2. Demandingess level of medication use and implementation methods of medication use at the unit    3. Medical services of the unit 3. Identification of risks related to MMU process and and means to manage the identified risks    1. Identification of risks related to MMU process of the unit and means to manage the identified risks    2. High-alert medicines of the unit    3. Medicines that mainly affect the central nervous system and medicines classified as narcotics, and prevention of medicine abuse situations    4. Actions taken by the unit in adverse event situations 4. Personnel responsibilities, duties, and division of labour in MMU process of the unit 5. Competence required for the implementation of MMU and ensuring competence of MMU 6. MMU process    1. Management of medication safety    2. Medication reconciliation and prescribing medicines    3. Procurement of medicines and the medication products selected to be used at the unit    4. Retain and disposal of medicines    5. Medicines owned by the patient (e.g., in situations where patient is in the hospital)    6. Medicine dispensing, compounding or preparation, double-checking and administration    7. Documentation of medication use at the unit    8. Monitoring the effects of medication treatment    9. Discontinuation of medication    10. Co-operation with other care units    11. Medication counselling of the patient and/or his relative 7. Follow up and feedback systems used at the unit    1. Follow up of adverse events related to medications    2. Reporting of adverse reactions of medicines and vaccines    3. Reporting of product defects and counterfeit of medicines    4. Reporting of adverse events related to medical devices    5. Patient feedback systems used    6. Instructing the patient and/or the relative about how to act in situations where problems in medication use are occurring    7. Medication safety audits conducted at the unit 8. Appendices of unit-based safe MMU protocol (e.g., MMU orientation form, unit-specific MMU operating and work instructions |
| --- |

Ministry of Social Affairs and Health. Safe pharmacotherapy : Guide to producing a pharmacotherapy plan [Internet]. Laukkanen E, Ruokoniemi P, editors. Sosiaali- ja terveysministeriö; 2021. Available from: <http://urn.fi/URN:ISBN:978-952-00-8682-4> [Abstract in English]
