# Supplementary material for: Medication Safety Risks and Their Management in Finnish Care Units: A Cross-Sectional Survey
Source: Health Serv Insights. 2026 Jul 23;19:11786329261472941. doi: 10.1177/11786329261472941 (PMC13396577; doi:10.1177/11786329261472941)
Supplement: Supplemental Material - Medication Safety Risks and Their Management in Finnish Care Units: A Cross-Sectional Survey [file sj-zip-1-his-10.1177_11786329261472941.zip › Supplementary_material3.docx]

Supplementary material 3. Study questionnaire. Questions reported in the present article are marked with asterix (*).

|  | **Question** | **Answer options** | **Response method** | **Transitions** |
| --- | --- | --- | --- | --- |
|  | **Section 1: Characteristics of respondents** | | | |
| * | 1. Education | Registered nurse  Bachelor of Pharmacy  Master of Pharmacy  Student | Choice of one option | As a result of answers “Registered nurse”, “Bachelor of Pharmacy” or “Master of Pharmacy”, moving to the question 3 |
| * | 1. Degree the respondent is studying (if the respondent is a student) | Registered nurse  Bachelor of Pharmacy  Master of Pharmacy  Other, which? | Choice of one option | Moving to the question 4 |
| * | 1. Year of completion of the stated education | Open text field | - | - |
| * | 1. Position at work | Manager  Employee | Choice of one option | - |
| * | 1. Gender | Female  Male  Other  Do not wish to answer | Choice of one option | - |
| * | 1. Age | Open text field | - | - |
| * | 1. Working environment | University hospital  Central hospital  Primary health care ward  Primary health care reception  Social care housing service  Home care  Other, which? | Choice of one option | - |
| * | 1. The location of the workplace | Southern Finland  Western Finland  Eastern Finland  Middle Finland  Northern Finland | Choice of one option | - |
| * | 1. Has a unit-based safe MMU protocol been developed at work unit (e.g., ward)? | Yes  No  I don’t know | Choice of one option | As a result of answers “No” and “I don’t know”, moving to the question 23 |
| * | 1. When was the last time you were familiarized with the unit-based safe MMU protocol of your unit? | Within a year  Over a year ago  Over two years ago  I haven’t read the unit-based safe MMU protocol of my unit | Choice of one option | As a result of answers “I haven’t read the unit-based safe MMU protocol of my unit” moving to the question 23 |
| * | 1. Have you participated in preparing and/or updating the unit-based safe MMU protocol of your unit? | Yes  No | Choice of one option | With the answer no moving on to the question 23 |
|  | **Section 2: Background information and policies on developing the unit-based safe MMU protocol** | | | |
| * | 1. When was the last update conducted for the unit-based safe MMU protocol of your unit (year)? | Open text field | - | - |
| * | 1. How often is the unit-based safe MMU protocol of your unit updated? | Every year  Every year or if necessary, when the practices changes  Every other year  Rarely  I don’t know | Choice of one option | - |
| * | 1. How has the nursing manager participated in developing or updating the unit-based safe MMU protocol? | Participated in writing  Participated in commenting  Approved the protocol  Not at all  I don’t know | Possibility to select several suitable options | - |
| * | 1. How have the nurses participated in developing or updating the unit-based safe MMU protocol? | Participated in writing  Participated in commenting  Approved the protocol  Not at all  I don’t know | Possibility to select several suitable options | - |
| * | 1. How have the physicians participated in developing or updating the unit-based safe MMU protocol? | Participated in writing  Participated in commenting  Approved the protocol  Not at all  I don’t know | Possibility to select several suitable options | - |
| * | 1. How have pharmacists participated in developing or updating the unit-based safe MMU protocol? | Participated in writing  Participated in commenting  Approved the protocol  Not at all  I don’t know | Possibility to select several suitable options | - |
| * | 1. A statement: The staff of our unit follows the unit-based safe MMU protocol, and the medication treatment instructions mentioned in it | Strongly agree  Agree  Neither agree nor disagree  Disagree  Strongly disagree | Choice of one option | - |
|  | **Section 3: Contents of the unit-based safe MMU protocol** | | | |
| * | 1. Evaluate how well the following areas are described in the unit-based safe MMU protocol of your unit    - Tasks and responsibilities of MMU for all professional groups (physicians, nurses, pharmacists, etc.)    - Competence required to implement MMU for all professional groups (physicians, nurses, pharmacists, etc.)    - Implementation of MMU orientation for all professional groups (e.g., physicianss, nurses, pharmacists, etc.)    - Providing in-house training related to MMU for staff    - Risks related to the MMU process of the unit (e.g., up-to-date information on the patient’s medication is missing or the patient is insufficiently identified when medication is administered, which may lead to medication being given to the wrong person)    - Practices of risk management (e.g., use of oral syringes in the unit that are not suitable for the IV route, medication reconciliation systematically conducted upon patient arrival at the unit)    - High-alert medicines in the unit (e.g., look alike sound alike medicines, high-alert medicines such as insulin, opioids, etc.)    - Risk management related to high-alert medicines    - Operation in incident situations (e.g., incorrect medicine administered to the patient)    - Practices and responsibilities for medication reconciliation upon patient arrival    - Prescribing practices    - Practices for dispensing and compounding or preparation of medicines and/or handling multi/unit dose-dispensing medicines    - Double-checking policies for medicines dispensed and compounded and/or multi/unit dose dispensing medicines    - Practices related to the administration of medicines (e.g., are same administration times always used at the unit, documenting medication administration information)    - Methods of identifying patients when administering medicines    - Monitoring the effects of medication treatment    - Practices and responsibilities for medication reconciliation when the patient is discharged or transferred to another care unit    - Giving medication counseling to the patient (who provides counseling and in which situations)    - Monitoring of the implementation of medication safety in the unit (e.g., quality indicators, patient safety indicators, ward inspections, error reports, etc.)    - Practices to ensure patient participation in the safe implementation of their own medication treatment (e.g., up-to-date medication list) | Fully described  Partially described  Planned to be described  Not described  I don’t know | Choice of one option | - |
|  | 1. What practical significance has the unit-based safe MMU protocol had in the development of the medication safety of the unit? | Open text field | - | - |
|  | 1. What challenges have there been in your unit in implementing practices in accordance with the unit-based safe MMU protocol? | Open text field | - | - |
|  | 1. What kind of support does your unit need or would have needed to implement practices that promote medication safety according to the unit-based safe MMU protocol? | Open text field | - | - |
|  | **Section 4: Implementation of practices related to medication safety** | | | |
|  | 1. Estimate how well the following practices related to medication safety are implemented in your unit (part 1/2)    - Responsibilities between professional groups in the implementation of MMU are clear    - A new employee (nurse, physician, pharmacist, etc.) receives a separate orientation to the implementation of MMU in the unit    - Only a person whose competence has been verified will participate in the MMU    - Our unit has organized training on the unit-based safe MMU protocol, and the practices described in it    - The staff regularly participates in in-house education related to MMU    - The staff knows the risks related to the MMU process of their own unit    - In our unit, protective measures have been taken to manage risks (e.g. use of oral syringes to avoid confusion of administration routes)    - The staff is familiar with the high-alert medicines in our unit    - To control the risks related with high-alert medicines, protective measures have been taken (e.g., keeping look alike sound alike medicines separately, limited selection in terms of strengths, warning labels)    - Reports of adverse events (e.g., Haipro system) are dealt with with the staff regularly    - Development measures are planned and implemented in relation to adverse event reports to prevent similar events    - The impact of agreed development measures on medication safety is monitored | Excellent  Good  Fair  Poor  Very poor  I don’t know | Choice of one option | - |
|  | 1. Estimate how well the following practices related to medication safety are implemented in your unit (part 2/2)    - Medication reconciliation is conducted when the patient enters the care unit    - Medicines that have been dispensed and compounded or prepared are marked with two different patient identifiers (e.g., name and date of birth). OBS! The patient location is not sufficient patient's identifier    - Double checking is carried out for ALL (e.g., pre-distributed, necessary medicines, injections) administered medicines    - The patient is always identified when medicines are administered    - The effects of pharmacotherapy are monitored regularly (e.g., use of a pain meter) and the information is recorded in the patient information system    - Patients are given advice and guidance when starting a new medication or when changing the medication    - Medical information sources based on researched information are used (e.g. Terveysportti, Duodecim medicine database, Pharmaca Fennica, etc.)    - Medication reconciliation is conducted when the patient leaves home or transfers to another care unit    - The patient actively participates in ensuring the safe implementation of their own pharmacotherapy (e.g., encouraged to share their own observations regarding pharmacotherapy)    - Medication safety audits are carried out in our unit (In the audit, a person outside the unit determines whether the medication use and management process of unit is safe and as described in the unit-based safe MMU protocol) | Excellent  Good  Fair  Poor  Very poor  I don’t know | Choice of one option | - |
|  | 1. At what level do you feel the medication safety culture in your unit is? | Excellent  Good  Fair  Poor  Very poor | Choice of one option | - |

MMU = medication management and use
