# Supplementary material for: Medication Safety Risks and Their Management in Finnish Care Units: A Cross-Sectional Survey
Source: Health Serv Insights. 2026 Jul 23;19:11786329261472941. doi: 10.1177/11786329261472941 (PMC13396577; doi:10.1177/11786329261472941)
Supplement: Supplemental Material - Medication Safety Risks and Their Management in Finnish Care Units: A Cross-Sectional Survey [file sj-zip-1-his-10.1177_11786329261472941.zip › Supplementary_material4.docx]

Supplementary material 4. Comprehensiveness of the unit-based safe MMU Protocol for Medication Safety Practices by the work environment.

| Sum variables | University hospital | Central hospital | Primary health care ward | Primary health care reception | Social care housing service | Home care | Other |
| --- | --- | --- | --- | --- | --- | --- | --- |
|  | mean SD | mean SD | mean SD | mean SD | mean SD | mean SD | mean SD |
| Total sum variable* |  |  |  |  |  |  |  |
| Coverage of the content of the unit-based safe MMU protocol in terms of medication safety practices (n=356) | 3.29 0.56 | 3.21 0.58 | 3.52 0.36 | 3.25 0.65 | 3.39 0.53 | 3.26 0.64 | 3.34 0.68 |
| Subtotal sum variable* |  |  |  |  |  |  |  |
| Competence, training, and orientation in pharmacotherapy (n=357) | 3.29 0.59 | 3.24 0.60 | 3.39 0.58 | 3.33 0.67 | 3.49 0.48 | 3.48) 0.54 | 3.38 0.61 |
| Risks associated with the pharmacotherapy process (n=357) | 3.31 0.69 | 3.21 0.66 | 3.49 0.51 | 3.23 0.79 | 3.36 0.63 | 3.10 0.87 | 3.25 0.81 |
| Prescribing, dispensing, and dosing practices (n=354) | 3.50 0.61 | 3.43 0.63 | 3.71 0.34 | 3.33 0.66 | 3.56 0.57 | 3.29 0.69 | 3.51 0.68 |
| Medication reconciliation and patient involvement (n=355) | 3.04 0.84 | 2.92 0.87 | 3.45 0.47 | 3.13 0.92 | 3.14 0.79 | 3.26 0.74 | 3.21 0.87 |
| Management of medication safety**(n=355) | 2.96 1.08 | 2.94 0.85 | 3.33 0.75 | 3.11 0.97 | 3.19 0.91 | 3.00 1.26 | 3.18 1.02 |
| * The sum variables were compiled for those respondents who had answered at least 70% of the statements in the respective sub-area (see Supplementary material 2, question 19)  ** The average of the subtotal sum variable has been calculated based on the responses to only one statement (Monitoring the implementation of the unit's medication safety; see Supplementary material 2, question 19). | | | | | | | |

MMU=Medication management and use

Kruskal-Wallis test was used for the evaluation of statistical significances. No statistically significant differences were observed.
