# Supplementary material for: Medication Safety Risks and Their Management in Finnish Care Units: A Cross-Sectional Survey
Source: Health Serv Insights. 2026 Jul 23;19:11786329261472941. doi: 10.1177/11786329261472941 (PMC13396577; doi:10.1177/11786329261472941)
Supplement: Supplemental Material - Medication Safety Risks and Their Management in Finnish Care Units: A Cross-Sectional Survey [file sj-zip-1-his-10.1177_11786329261472941.zip › Supplementary_material5.docx]

Supplementary material 5. Means and standard deviations of statements describing the content of the unit-based safe MMU protocol

| **Statements of question 19** | **N** | **Mean** **(1-4)** | **SD** |
| --- | --- | --- | --- |
| Duties and responsibilities of MMU | 361 | 3.72 | 0.57 |
| Competence required for the implementation of MMU | 357 | 3.64 | 0.60 |
| Implementation of MMU orientation | 356 | 3.30 | 0.80 |
| Provision of in-house training in MMU | 355 | 2.77 | 1.10 |
| Risks associated with the MMU process of unit | 352 | 3.38 | 0.82 |
| Risk management tools | 357 | 3.04 | 1.07 |
| High-alert medicines of unit | 355 | 3.36 | 0.90 |
| Risk management of high-alert medicines | 355 | 3.20 | 0.93 |
| Operation in incident situations | 356 | 3.54 | 0.83 |
| Medication reconciliation practices upon arrival | 352 | 3.33 | 0.94 |
| Prescribing practices | 355 | 3.62 | 0.76 |
| Distribution and compounding or preparation of medicines | 355 | 3.68 | 0.70 |
| Double-checking policies | 357 | 3.57 | 0.83 |
| Administration practices | 355 | 3.46 | 0.91 |
| Patient identification methods | 357 | 3.41 | 0.95 |
| Monitoring the effects of pharmacotherapy | 351 | 3.25 | 0.89 |
| Medication reconciliation practices on departure | 356 | 3.15 | 1.05 |
| Providing guidance and counseling on medication | 356 | 3.07 | 1.04 |
| Monitoring the implementation of medication safety | 356 | 3.09 | 0.95 |
| Ensuring patient involvement | 353 | 2.96 | 1.11 |

MMU= medication management and use
